# B7H6 Serves as a Negative Prognostic Marker and an Immune Modulator in Human Pancreatic Cancer

**DOI:** 10.3389/fonc.2022.814312

**Published:** 2022-03-03

**Authors:** Zheng Zhu, Kun-Yu Teng, Jian Zhou, Yunyun Xu, Lifeng Zhang, Hua Zhao, Xueguang Zhang, Lei Tian, Zhiyao Li, Ting Lu, Shoubao Ma, Zhenlong Li, Zhenyu Dai, Jing Wang, Xingyu Chen, Xing Wu, Yihan Pan, Weiqiang Shi, Zhiqun You, Hanyu Chen, Vincent Chung, Jianhua Yu, Songbing He, Xin Zhao, Lei Cao, Dechun Li

**Affiliations:** ^1^ Department of General Surgery, The First Affiliated Hospital of Soochow University, Suzhou, China; ^2^ Department of Hematology and Hematopoietic Cell Transplantation, City of Hope National Medical Center, Los Angeles, CA, United States; ^3^ Jiangsu Institute of Clinical Immunology, The First Affiliated Hospital of Soochow University, Suzhou, China; ^4^ Jiangsu Key Laboratory of Clinical Immunology, Soochow University, Suzhou, China; ^5^ Jiangsu Key Laboratory of Gastrointestinal Tumor Immunology, The First Affiliated Hospital of Soochow University, Suzhou, China; ^6^ Pediatric Clinical Research Institute, Children’s Hospital Affiliated to Soochow University, Suzhou, China; ^7^ Department of General Surgery, Taizhou Fourth People’s Hospital, Taizhou, China; ^8^ Department of General Surgery, The First People’s Hospital of Huzhou, Huzhou, China; ^9^ College of Liberal Arts, University of Minnesota Twin Cities, Minneapolis, MN, United States; ^10^ Department of Pathology, The First Affiliated Hospital of Soochow University, Suzhou, China; ^11^ Department of Medical Oncology, City of Hope National Medical Center, Los Angeles, CA, United States

**Keywords:** B7 homolog 6 (B7H6), pancreatic cancer (PC), NK cells, NKp30, prognosis

## Abstract

Pancreatic cancer (PC), the third leading cause of cancer-related death in the U.S., is frequently found too late to be cured by traditional chemotherapy. Expression of B7 homolog 6 (B7H6), a member of the B7 family of immunoreceptors, has been found in PC and several other cancers. B7H6 is a ligand for cytotoxicity triggering receptor 3 (NKp30), which is expressed on NK cells. Here, we demonstrate that B7H6 can be detected in PC tissues but not normal organs. Its expression in patients associated significantly with tumor differentiation grade and lymphatic metastasis. The soluble form of B7H6 was detected in the PC patients’ sera, and its concentration associated with tumor differentiation grade and tumor, node, metastasis (TNM) stages. Also, higher levels of B7H6 in PC patients’ malignant tissues or serum correlated with shorter overall survival. *In vitro*, downregulation of B7H6 by CRISPR/Cas9 or siRNA technology had no significant impact on the viability or mobility of PC cells. Instead, knocking out B7H6 sensitized PC cells to NK-mediated cytotoxicity and cytokine production. These results indicate that B7H6 not only serves as a negative prognostic marker but also acts as an immune modulator in PC.

## Introduction

Pancreatic cancer (PC) is one of the most lethal human solid tumors, and patients’ 5-year overall survival rate remains stagnant at 7% to 9% (1). Thus, it is the third leading cause of cancer-related deaths in the United States and the seventh leading cause globally. In addition, PC is expected to be the third leading cause of cancer death globally by 2025 (2). There were 496,000 cases and 466,000 deaths globally in 2021, highlighting PC’s high fatality rate (2). As patients seldom exhibit symptoms at an early stage, 80% have locally advanced disease or distant metastasis at the time of diagnosis (3, 4). Although radical resection is a potential cure for PC (and the only cure as yet) (5), more than 90% of patients who undergo resection experience tumor relapse and die within 5 years (6). In recent decades, researchers have explored many treatment options, including chemotherapy, chemoradiation, and targeted therapies for PC. However, no treatment to date has remarkably improved the prognosis for PC patients (7, 8). Therefore, the need for novel diagnostic and treatment modalities is extremely urgent.

The success of immunotherapy is at the forefront of emerging cancer research. Many investigators are trying to find effective immunotherapeutic strategies for PC, including bispecific antibodies (Bi-Abs), immune checkpoint inhibitors, cancer vaccines, chimeric antigen receptor (CAR) T cells, combination therapies with immunotherapeutic agents, chemoradiotherapy, or other molecularly targeted agents. Although none of those has shown promising results (9), we expect further studies of interactions between immune cells and cancer cells to suggest new immunotherapeutic approaches.

We focused on homologue 6 (B7H6), also known as natural killer cell cytotoxicity receptor 3 ligand 1 (NCR3LG1), because it was newly discovered and found on several types of tumor cells. B7H6 is member of the B7 family (10), which also contains the apoptotic ligand PD-L1. B7H6 helps regulate innate immune responses by binding to an immune receptor, NKp30, which natural killer (NK) cells express (11).

NK cells are the immune system’s first line of defense against pathogens and cancers (12). These lymphocytes regulate both innate and adaptive immune responses by secreting pro-inflammatory cytokines and chemokines; they also mediate cytotoxic activity by directly lysing target cells. NK cells express activating receptors on their surface, such as NKp30, NKp44, and NKp46, that help recognize and eliminate abnormal target cells (13).

There are two forms of B7H6—cell-surface B7H6 and soluble B7H6 (sB7H6)—and both have been detected in primary tumors and the tumor microenvironment, including in glioma, neuroblastoma, breast cancer, non-small cell lung cancer, hepatocellular carcinoma, gastric carcinoma, ovarian cancer, leukemia, and lymphoma—though rarely in normal tissues (14–22). Cell- surface B7H6 associates with cell damage, and it triggers innate immunity when it binds to NKp30 on NK cells (23). However, sB7H6 can decrease NKp30 expression on NK cells and induce NK cell dysfunction in primary as well as metastatic cancer (24).

In this study, we evaluated the expression of B7H6 in the pancreas, using surgical specimens from 66 PC patients. Immunohistochemistry (IHC) detected B7H6 expression in primary tumor tissue as well as in adjacent tissue but not in normal healthy organ sections. Furthermore, expression correlated significantly with tumor differentiation grade and lymphatic metastasis. Levels of sB7H6 were also higher in sera from an additional 65 PC patients than in sera from healthy donors. Moreover, concentration of sB7H6 showed strong correlation with tumor differentiation grade and tumor, node, metastasis (TNM) stage. Overall, PC patients with higher expression of B7H6 in either malignant tissue or sera had poorer overall survival.

To understand the effects of B7H6 in PC tumor cells, we conducted functional studies in pancreatic cancer cell lines, using CRISPR/Cas9 or siRNA technology. Knocking out B7H6 in PC cells did not alter cell viability or mobility. However, co-culturing knocked-out B7H6 tumor cells with NK cells *in vitro* significantly enhanced the NK cells’ cytolytic function and cytokine production. These results indicate that B7H6 might be a negative prognostic marker that modulates NK cell function in PC.

## Materials and Methods

### Patient Characteristics and Preparation of Patient Samples

Clinical data were collected from pancreatic cancer patients between September 2010 and December 2015 in The First Affiliated Hospital of Soochow University in Suzhou, China. These patients had been diagnosed pathologically and had undergone surgery, but they had not received any chemotherapy or radiotherapy. Pathological tissue samples from 66 initial patients and serum from an additional 65 patients and 34 healthy donors were analyzed for expression of B7H6 by IHC or ELISA, respectively. To analyze 15 nonmalignant samples, we used tissues collected from patients without PC: brain tissue adjacent to a pilocytic astrocytoma; heart tissue adjacent to a hamartoma of mature cardiac myocytes; thyroid tissue from a patient with follicular adenoma; placental tissue from women who had just given birth, and various tissues from injury cases. The protocol for the study was approved by The First Affiliated Hospital of Soochow University.

### Cell Lines and Cell Culture

The human pancreatic cancer cell lines CFPAC-1 and PL45 were purchased from the Chinese Academy of Science Cell Bank. The human pancreatic cancer cell lines Capan-1 and PANC-1 were a gift from Dr. Yuan Chen (City of Hope). The human erythroleukemic cell line K562 was purchased from the American Type Culture Collection (ATCC). Capan-1 and CFPAC-1 cells were cultured in IMDM with 20% fetal bovine serum (FBS) and 1% antibiotic (Thermo Fisher Scientific, USA). MiaPaCa-2, PL45, and PANC-1 cells were cultured in DMEM with 10% FBS and 1% antibiotic. K562 cells were cultured in RPMI 1640 medium supplemented with 10% FBS and 1% antibiotic. All the cell lines were incubated under standard culture conditions (37°C and 5% CO_2_). PRMI 1640, DMEM, IMDM, and FBS were purchased from Gibco (Cambrex, MD, USA).

### NK Cell Isolation and Culture

Cord blood leukopaks were obtained from StemCyte. Primary NK cells were enriched from cord blood, using RosetteSep (StemCell Technologies) and following the manufacturer’s instructions (25). Primary NK cells were cultured in RPMI 1640 with 5% AB sera and 50 IU/mL IL-2.

### Flow Cytometry

Cells were resuspended in fluorescence-activated cell sorting (FACS) buffer composed of phosphate-buffered saline (PBS; Gibco), 2 mM EDTA (Gibco), and 2% FBS (Gibco). They were then stained with B7H6 (eBioscience, clone: JAM1EW), NKp30 (Biolegend, clone: P30-15), HLA-A.B.C (Biolegend, clone:W6/32), NKG2D (BD Pharmingen™, clone: 1D11), NKp46 (BD Pharmingen™, clone: 9-E2), NKG2A (Miltenyi Biotec, clone: REA 110), PD-1 (BD Horizon™, clone: MIH4), TIGIT (BD OptiBuild™, clone: 741182), CD69 (BD Pharmingen™, clone: FN50) and their isotype control at room temperature in the dark for 30 minutes. The cells were washed two times with FACS buffer, and then analyzed with a Fortessa X20 flow cytometer (BD Biosciences). To examine expression levels of intracellular TNF-α, granzyme B, and perforin, we co-cultured 1.0 × 10^5^ NK cells with tumor supernatants and 50 U/mL recombinant human IL-2, 100 μg/mL streptomycin, and 100 U/mL penicillin in 96-well flat-bottomed plates. GolgiStop Protein Transporter Inhibitor (BD Biosciences, San Jose, CA) was added to the medium for the staining. Plates were incubated at 37°C overnight in a humidified atmosphere of 5% CO_2_. NK cells were harvested and stained with TNF-α, granzyme B, and perforin (BD Biosciences) antibodies, using an eBioscience™ Intracellular Fixation & Permeabilization Buffer Set (BD Biosciences). The percentage of cells positive for each parameter was determined with a Fortessa X20 flow cytometer (BD Biosciences).

### Immunohistochemistry

Pancreatic tissue blocks were cut into 5-μm-thick sections, dewaxed in xylene, and rehydrated in in an ethanol gradient. Antigen was retrieved by boiling the tissue sections for 20 minutes in the retrieval buffer. Sections were later immersed in a 3% hydrogen peroxide solution for 15 min to block endogenous peroxidase activity. Next, the slides were rinsed with PBS 3 times, blocked with 3% BSA at room temperature for 30 minutes, and then incubated with purified rabbit anti-human primer antibody (1:300 dilution) at 4 °C overnight. After the incubation, the slides were incubated with diluted goat anti-rabbit secondary antibody for 1 hour at room temperature. They were then rinsed twice with PBS. The detection reagent DAB was added, and the slides were incubated in the dark at room temperature for 10 minutes. After DAB, the slides were rinsed in running tap water for 3 minutes. Finally, they were incubated with hematoxylin to counterstain the nucleus. All slides were independently examined by two authorized pathologists who were not informed of the patients’ clinical statuses and outcomes (26). B7H6 immunostaining densities were assessed according to the H-score method. The H-score was calculated as follows: (% tumor cells unstained x0) + (% tumor cells weakly stained x1) + (% tumor cells moderately stained x2) + (% tumor cells strongly stained x3), with the score ranging from 0 (100% negative tumor cells) to 300 (100% strongly staining tumor cells). The pathologists analyzed five zones of the same section.

### Enzyme-Linked Immunosorbent Assay (ELISA) for B7H6 and IFN-γ

sB7H6 from patients’ sera and cell culture supernatants was analyzed with a Human B7-H6 DuoSet ELISA kit (DY7144-05; R&D) following the manufacturer’s protocol. IFN-γ was detected in cell culture supernatant and analyzed with commercially available mAb pairs (Thermo Fisher Scientific, Rockford, IL, USA) according to the manufacturer’s protocol, as previously described (27).

### Immunoblotting

To assess expression of B7H6 protein, we first homogenized whole cells or patients’ pancreatic tissue in 10 volumes of RIPA lysis buffer containing (in mM) 1 PMSF, 1 DTT, and 0.02% (v/v) protease cocktail (Sigma Aldrich, St. Louis, MO, USA). The homogenates were centrifuged at 20,000×g at 4°C for 15 min, and the supernatants were retained. Protein concentrations were determined by the BCA method. Equal amounts of protein from each sample were loaded onto SDS–PAGE gel and transferred to nitrocellulose membranes. The membranes were blocked with 3% BSA/TBS-T for 1 hour at room temperature and then incubated with B7H6 antibody (MAB7144, Minneapolis) or anti-glyceraldehyde 3-phosphate dehydrogenase (GAPDH) antibody (ab9484, Abcam) in 1% BSA/TBS-T overnight at 4 °C. Following washing, the nitrocellulose membranes were incubated with a specific horseradish peroxidase-conjugated secondary antibody (DAKO, 1:5000) for 1 hour at room temperature, washed with PBS, and visualized with ECL (Thermo Fisher). Signals were detected with the FluorChem E system (Protein Simple, San Jose, CA). Each band was normalized to an internal control and quantified with ImageJ software.

### RT-PCR and Quantitative PCR (RT-qPCR)

Total RNA was extracted using an RNeasy Plus Mini Kit (74034, Qiagen). It was then reverse-transcribed to cDNA by using SuperScript VILO Master Mix (11755050; Invitrogen) and following the manufacturer’s instructions. The cDNA was amplified by PCR, and the products were visualized under the Bio-Red gel documentation system. Quantitative PCR was performed using SYBR Green (A25741, Waltham) and the QuantStudio™ 7 Flex Real-Time PCR System (4485701, Thermo Fisher) following the manufacturer’s instructions.

### B7H6 Knockdown Mediated by siRNA

To knock down B7H6 in PC cells, we used siRNA targeting the B7H6 gene or scramble siRNA, which were purchased from Integrated DNA Technologies, Inc. (hs.Ri.NCR3LG1.13, IDT). Lipofectamine 3000 was used to deliver the siRNAs into the pancreatic tumor cells. (L3000001, Thermo Fisher). Briefly, the human pancreatic cancer lines Capan-1 and PANC-1 were seeded into 24-well plates at a density of ~5 × 10^4^ cells/well one day prior to transfection. The siRNAs were delivered to the cells at approximately 60% confluence. Twenty-four hours after transfection, the efficiency of each siRNA was analyzed by qPCR and immunoblotting.

### Cell Proliferation Analysis by CCK-8 and RTCA

The proliferation of PC cells was evaluated with a Cell Counting Kit-8 (C0038, Beyotime) following the manufacturer’s instruction. Briefly, 5x10^4^ Capan-1 and PANC-1 cells transfected with B7H6-siRNA or scramble siRNA were seeded separately into wells of a 96-well plate and cultured in 100 μL of cell culture media. At the indicated time points, the media were replaced with CCK-8 reagent (10 μL CCK-8 and 90 μL Media), and the cells were incubated for an additional hour. Absorbance of each well was then measured at 450 nm to determine cell growth, which was monitored every 24 hours over 3 days. For real time cell analysis (ACEA Bioscience, xCELLigence RTCA MP), as reported earlier (28), PC cells were seeded at a density of 5,000 cells/well and incubated in an xCELLigence RTCA MP instrument at 37°C for 72 hours. Data were collected every 15 minutes for a total of 72 hours. The cells’ normalized cell index was analyzed using xIMT software (ACEA Biosciences Version 2.3.2). Cells were also seeded into 6-well plates at 1×10^5^ cells per well and incubated for 72 hours so that cell morphology could be observed with a microscope.

### Apoptosis Assay

Cell apoptosis was examined with an APC Annexin V Apoptosis Detection Kit (88-8007-72, Thermo Fisher) following the manufacturer’s instructions (29). Briefly, Capan-1 and PANC-1 cells from the groups transfected with B7H6-siRNA or scramble siRNA were harvested. Then 1×10^6^ cells from each group were washed with PBS, resuspended in 100 μL binding buffer, and incubated with 5 μL APC Annexin V and 5 μL propidium iodide (PI) solutions. After a 20-minute incubation in the dark, the cells were analyzed with a Fortessa X20 flow cytometer (BD Biosciences).

### Generating B7H6-Knockout Cells With CRISPR/Cas9

To knock out B7H6 in pancreatic tumor cells, we performed CRISPR/Cas9 genome editing, using the all-in-one plasmid (06182015MN, Sigma-Aldrich). Briefly, Capan-1 and PANC-1 cells were transfected with the CRISPR plasmids, using Lipofectamine 3000 (L3000001, Thermo Fisher). Puromycin (10 μg/mL) (P8833-25MG Sigma-Aldrich) was used subsequently to select the edited cells. Puromycin-resistant cells were then FACS-sorted into singlets, using a BD Aria Fusion flow cytometer. The edited cells were then expanded and verified by immunoblotting analysis.

### Wound Healing Assay

Cell migration was evaluated with the wound healing assay. Briefly, wild type and B7H6 KO pancreatic tumor cells were incubated in 6-well plates. Using the tip of a 200 μL pipette, we then made a small lengthwise stripe in the 90% confluent monolayer. The wounded cells were then washed twice with PBS and further incubated in serum-free DMEM at 37°C in a 5% CO_2_ incubator for an additional 24 hours. At the indicated time points, we photographed the cells and measured the wound width at 100X magnification, using a Zeiss fluorescence microscope (AXIO observer 7). Ten measurements were made at random intervals along the length of the wound, and the data were averaged to express them as percentages of the wound’s original width. The experiment was performed in triplicate and repeated at least twice.

### Transwell Invasion Assay

The Transwell system was used to evaluate the invasive ability of Capan-1 and PANC-1 cells. In brief, the upper chamber (8µm) was coated with 20 μL Matrigel diluted 1:4 in serum-free media and incubated at 37 °C for 4 hours. Then Capan-1 and PANC-1 cells were seeded into the medium at a density of 2x10^5^/200 μL. The lower chamber was filled with 600 μL culture medium supplemented with FBS as a chemoattractant. After 24 hours of incubation, the non-invading cells and the Matrigel were removed from the upper chamber. The insert was rinsed with PBS, and the invaded cells on the filters were fixed with methanol for 30 minutes and stained with crystal violet (Sigma-Aldrich). The number of invading cells was counted in 6 random fields per filter at 100X magnification, and the counts were performed three times.

### Flow-Based Cytotoxicity Assay

Target cells were harvested and resuspended at a dilution of 8x10^4^/mL. Then 100 μL of the cell suspension was added to an Ultra-Low Attachment Multiple Well Plate (CLS3474, Corning). This suspension was co-cultured with effector cells (primary human NK cells) enriched from cord blood (StemCyte) at an effector/target (E/T) ratio of 10:1 at 37°C in a 5% CO_2_ atmosphere for 24 hours. The cytotoxicity of the NK cells was then evaluated with an ACEA NovoCyte Flow Cytometer.

### Determining NK Cell Cytotoxicity With the Chromium^51^ Release Assay

A standard 4-h chromium^51^ release assay was performed as described previously (30, 31). For the soluble B7H6 blocking assay, the blocking antibody (R&D Systems, MAB71444) at 100 ng/mL was preincubated with the supernatants for 40 minutes and then co-cultured with NK cells for 4 hours. The percentage of specific cell lysis was calculated using the standard formula: 100 × (cpm experimental release – cpm spontaneous release)/(cpm maximal release – cpm spontaneous release).

### Statistical Analyses

Continuous endpoint results are presented as mean ± SD assuming all data are normally distributed. For data with repeated measures from the same subject/donor, a linear mixed model was used to account for the underlying variance and covariance structure. Statistical analyses were performed using the GraphPad, R.3.6.3. software package (GraphPad Software, Inc., San Diego, USA). A paired Student’s *t*- test, Wilcoxon rank test, and Chi-square test was used as appropriate. A *P*-value of <0.05 was considered statistically significant.

## Results

### Correlations Between B7H6 Expression and Clinical Characteristics in PC Patients’ Surgical Specimens

**Figure 1:**
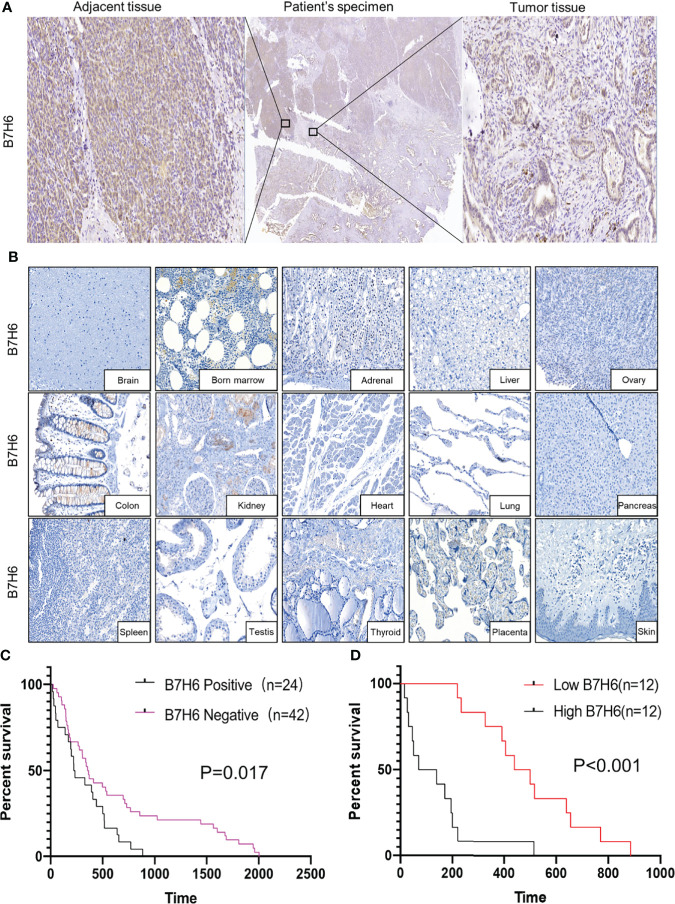
B7H6 expression in human pancreatic cancer tissues. **(A)** IHC staining of B7H6 in tumor (right) and adjacent tissues (left) of a pancreatic cancer patient’s specimens (magnification: Middle, 10×; right and left, 100×). **(B)** B7H6 expression in benign human organs shown by IHC staining of B7H6 (magnification: 100×). **(C)** Kaplan-Meier survival analysis of overall survival between PC patients who carried B7H6-positive or -negative tumors. *P*=0.017. **(D)** Kaplan-Meier analysis of overall survival between patients with high levels of B7H6 (H score ≥ 47) and those with low levels (H-score < 47). *P*<0.001.

To obtain expression profiles, we used IHC to analyze B7H6 expression in PC specimens from 66 PC patients and 15 organ tissues from nonmalignant patients. We detected B7H6 expression on the pancreatic cancer tissues as well as on adjacent tissues in 24/66 (36.4%) of the patients’ samples (**Figure 1A**). The median H-score for B7H6 expression was 47 (12–210). Of note, we did not find B7H6 expression in the normal tissues except for weak staining in the kidney (**Figure 1B**). The differential expression of B7H6 in pancreatic cancer tissue compared to normal pancreas is shown in **Supplementary Figure 1A**. To verify this difference, we used immunoblotting to assess B7H6 expression in 11 pairs of tumor tissues and adjacent tissues. Both primary tumors and adjacent tissues expressed B7H6 (**Supplementary Figure 1B**). When we examined correlations between B7H6 expression and clinical characteristics, we found that expression in patients’ tumor tissues associated significantly with differentiation grade (*P*=0.034) and lymphatic metastasis (*P*=0.035) but not with other clinical parameters (**Table 1**). Information about those 66 patients is summarized in **Supplementary Table 1**. To explore the prognostic importance of B7H6 expression in PC patients, we performed Kaplan-Meier analysis, using the log-rank test to estimate survival. The patients with positive B7H6 expression had statistically significantly shorter survival compared to the B7H6-negative patients (*P*=0.017, **Figure 1C**). We then divided the patients with B7H6-positive tumors into two groups: high vs. low B7H6 expression (H-score). The high-B7H6 group survived for a significantly shorter time than the low-B7H6 group (*P*<0.0001, **Figure 1D**), suggesting that B7H6 expression in tumors correlates strongly with poor prognosis in PC patients.

**Table 1:** Clinical significance of B7H6 expression on tissues.

B7H6 expression level						
Clinical parameters	Cases	0 < H-score < 47	H-score ≥ 47	H-score = 0	χ2	*P*-value
**Gender**					**1.627**	**0.443**
Male	40	9	6	25		
Female	26	3	6	17		
**Age (years)**					**3.102**	**0.212**
<68	31	3	7	21		
≥68	35	9	5	21		
**Tumor size (cm)**					**1.122**	**0.571**
<2.0	20	5	4	11		
≥2.0	46	7	8	31		
**Tumor location**					**0.354**	**0.838**
head	39	7	8	24		
body and/or tail	27	5	4	18		
**Differentiation grade**					**10.390**	0.034*
Low	11	1	5	5		
Middle	51	9	6	36		
High	4	2	1	1		
**Lymphatic metastasis**					**6.706**	0.034*
Yes	25	1	7	17		
No	41	11	5	25		
**TNM stages**					**0.224**	**0.894**
I+II	29	6	5	18		
III+IV	37	6	7	24		

*P < 0.05. Bold means that P-value <0.05 was considered significant.

### Correlations Between sB7H6 Expression in Sera and PC Patients’ Clinical Characteristics

**Figure 2:**
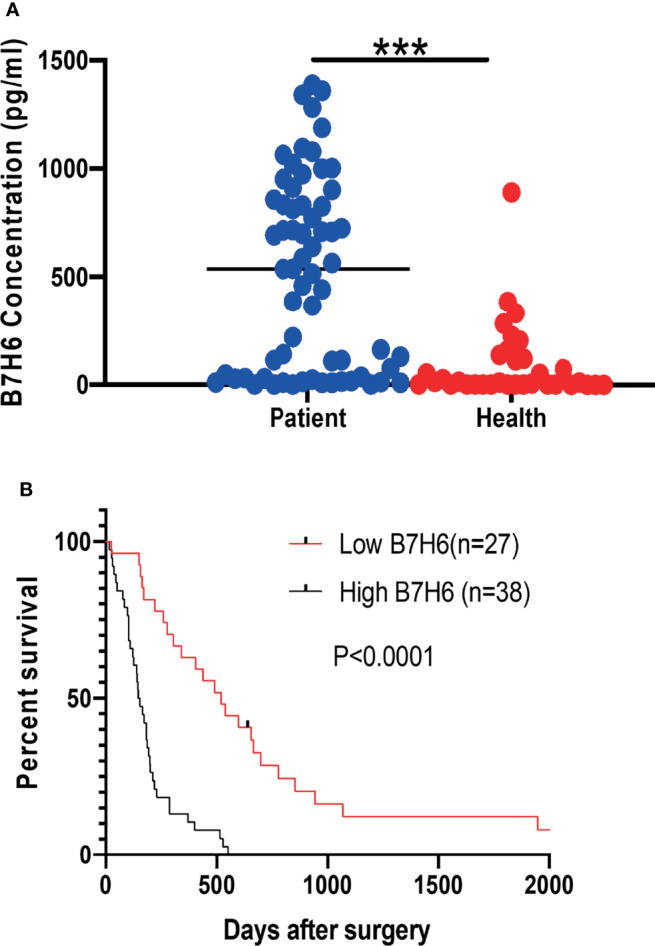
Relationship between soluble B7H6 expression in serum and prognosis for PC patients. **(A)** sB7H6 levels in serum of PC patients (n=65) and healthy donors (n=34). Data are from at least 3 independent experiments. **(B)** Kaplan-Meier analysis of overall survival of PC patients grouped by B7H6 levels, using the median (219.5pg/mL) as a cut-off (***P < 0.001).

To understand the clinical implications of sB7H6, we collected sera from an additional 65 PC patients and 34 healthy volunteers. sB7H6 levels were determined with an enzyme-linked immunosorbent assay (ELISA). Serum concentrations of sB7H6 were significantly higher in the PC patients than in the healthy donors (505.3± 440.5 pg/mL versus 88.45 ± 174.3 pg/mL, *P*< 0.0001, **Figure 2A**). Next, we further divided the PC patients into high-sB7H6 and low-sB7H6 groups, using the median concentration (219.5pg/mL) as the cut-off. Then we looked for correlations between sB7H6 concentration and clinical characteristics. Higher levels of sB7H6 in sera correlated significantly with lower differentiation grade (*P*=0.0378) and later TNM stage (*P*<0.001) but not with any of the other clinical parameters we included (**Table 2**). To determine whether sB7H6 level associated with poor prognosis in PC, we performed survival analysis using the log-rank test. The patients with higher sB7H6 levels had significantly shorter overall survival compared with those with lower sB7H6 levels (*P*<0.0001, **Figure 2B**). These data further imply that sB7H6 could serve as a negative prognostic marker for pancreatic cancer patients.

**Table 2:** Clinical significance of sB7H6 expression.

sB7H6 expression level					
Clinical parameters	Cases	ELISA<=219.5	ELISA>219.5	χ2	*P*-value
**Gender**				**0.831**	**0.362**
Male	38	14	24		
Female	27	13	14		
**Age (Years)**				**0.376**	**0.54**
<60	19	9	10		
≥60	46	18	28		
**Tumor size (cm)**				**3.827**	**0.05**
<2.0	12	8	4		
≥2.0	53	19	34		
**Tumor location**				**0.367**	**0.545**
head	43	19	24		
body and/or tail	22	8	14		
**Differentiation grade**				**6.550**	0.0378*
Low	20	4	16		
Middle	41	20	21		
High	4	3	1		
**Lymphatic metastasis**				**0.004**	**0.95**
Yes	31	13	18		
No	34	14	20		
**TNM stages**				**15.339**	0.00009***
I+II	21	16	5		
III+IV	44	11	33		

*P<0.05, ***P<0.001. Bold means that P-value <0.05 was considered significant.

### Downregulation of B7H6 Does Not Affect the Proliferation, Apoptosis, or Mobility of Pancreatic Cancer Cell Lines

**Figure 3:**
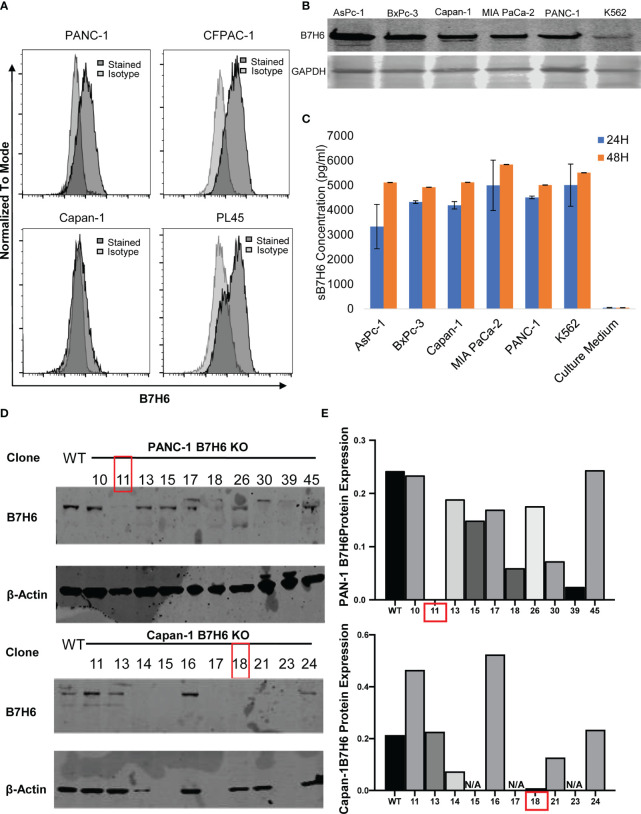
B7H6 expression in human PC cell lines and generation of B7H6 KO PC cell lines. **(A)** Surface expression of B7H6 on human pancreatic cancer cell lines (PANC-1, CFPAC-1, Capan-1, PL45) was assessed with a Fortessa X20 flow cytometer. **(B)** Immunoblotting detected B7H6 protein expression in human pancreatic cancer cell lines, with K562 as a positive control. GAPDH was used as a loading control. **(C)** sB7H6 secreted by pancreatic cancer cells into supernatants at 24 and 48 hours was analyzed by ELISA. Medium collected from K562 or RPMI-1640 served as a positive or negative control, respectively. Data from at least 3 independent experiments are shown as mean ± SEM. **(D)** Immunoblotting detected the protein expression of B7H6 after *what* was knocked out by CRISPR/Cas9 from PANC-1 and Capan-1 cells. β-actin was the internal control **(E)** Quantification of B7H6 protein expression on PANC-1 and Capan-1 cells normalized to β-actin.

When we evaluated B7H6 expression in several human PC cell lines (using a Fortessa X20 flow cytometer), we detected it on the cell surface of PANC-1, CFPAC-1, and PL45 cells but not on Capan-1 cells (**Figure 3A**). However, immunoblotting detected B7H6 on all the pancreatic cancer cell lines, including Capan-1 (**Figure 3B**). The discrepancy could reflect a difference between flow cytometry and immunoblotting or it could indicate that Capan-1 cells express only soluble B7H6 and not cell-surface B7H6. The latter explanation might be valid, as ELISA identified B7H6 in the cell culture supernatants of all the PC cells, including the K562 cells used as a technical control (**Figure 3C**). These data suggest that B7H6 might not be expressed exclusively on the cell surface, at least in some PC cell lines, but might instead be retained in the cytosol or released into the supernatants of cultured cells.

To investigate the role of B7H6 in PC cell lines, we knocked it down, using siRNA; then we confirmed knockdown efficiency by qPCR and immunoblotting (**Supplementary Figures 2A-C**). To assess whether B7H6 knockdown impacted tumor growth, we used the Cell Counting Kit-8 (CCK-8). The proliferation of PC cells with scramble siRNA or B7H6 knockdown did not differ significantly (**Supplementary Figures 2D, E**). We next evaluated whether B7H6 knockdown induced apoptosis in PC tumor cells by analyzing the expression of [ … by staining with]??? annexin V/propidium iodide. Flow cytometry demonstrated that knocking down B7H6 had no effect on PC cell apoptosis compared to scramble siRNA demonstrated that neither B7H6 siRNA nor scramble siRNA promoted apoptosis (**Supplementary Figure 2F**).

**Figure 4:**
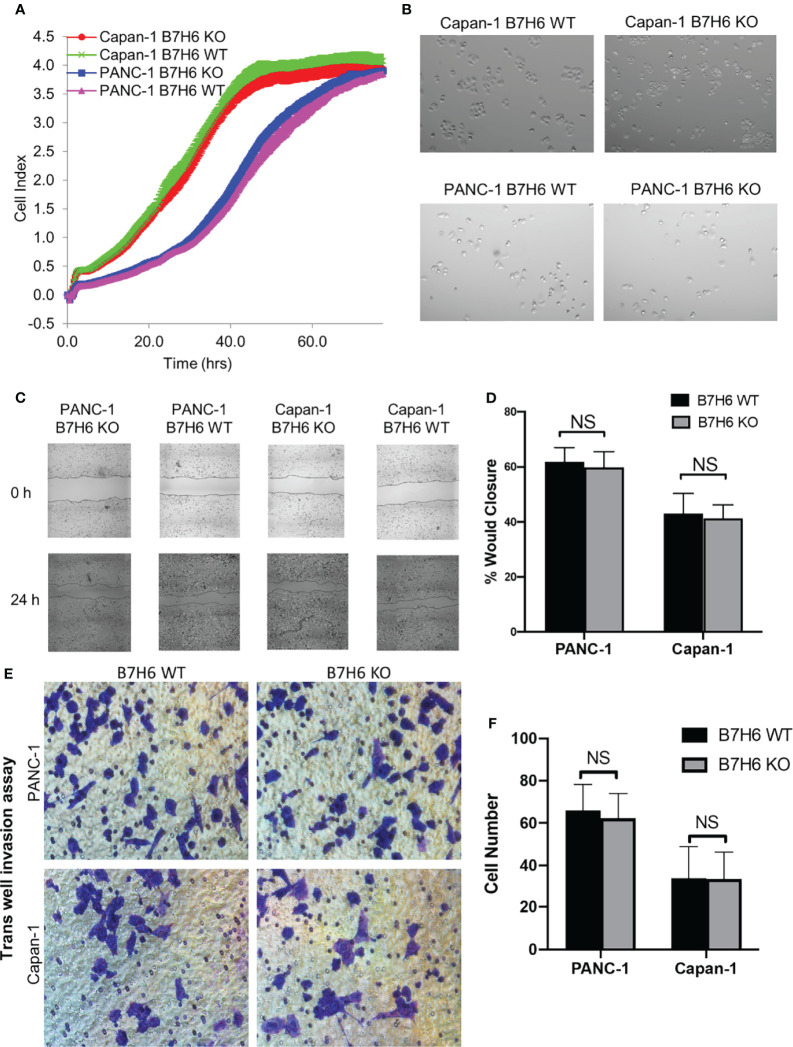
Knocking down B7H6 does not affect cell survival, apoptosis, or migration of pancreatic cancer cell lines. **(A)** RTCA analysis of proliferation of WT and B7H6 KO PC cells. The x-axis shows the time of the assay. The y-axis represents growth (fold change) of the indicated cells. **(B)** Cell morphology images of WT or B7H6 KO PC cells under a microscope at 100x magnification. **(C)** Representative image from a wound healing assay performed with B7H6 WT and KO of PANC-1 or Capan-1 cells. **(D)** The wound healing assay was quantified using ImageJ. **(E)** Transwell invasion assay of B7H6 WT and KO of PANC-1 and Capan-1 cells (magnification 200×). **(F)** Quantification of invaded cells from the Transwell assay. Data from at least 3 independent experiments are shown as mean ± SEM. NS, not significant

To investigate potential functions of B7H6 in PC tumor cells, we used qPCR to check the expression of several genes associated with PC: CD133, HIF1a, MYC, OCT4, VEGF, and ZEB2 (32–37). Expression of these genes did not differ significantly between B7H6 siRNA knockdown cells and scramble siRNA cells (**Supplementary Figure 3**). To verify the data from the siRNA experiment, we generated B7H6 knockout (KO) PANC-1 and Capan-1 cell lines by CRISPR/Cas9 genome editing. Several B7H6 KO clones of PANC-1 and Capan-1 cell lines were validated by immunoblotting (**Figures 3D, E**). As clone 11 of PANC-1 and clone 18 of Capan-1 showed negligible B7H6 expression, we selected them for functional validation. (Of note, both the cell-surface form and the soluble form of B7H6 were knocked out in these two clones). When we used real-time cell analysis (RTCA) or a microscope to compare cell proliferation, we found no significant difference between the B7H6 KO and wild type (WT) cells (**Figures 4A, B**). Wound healing analysis (**Figures 4C, D**) and Transwell invasion (**Figures 4E, F**) also revealed no significant differences between B7H6 WT and KO cells. Collectively, these data suggest that B7H6 may not directly impact cell proliferation, apoptosis, or migration of PC cells.

### Knocking out B7H6 From Pancreatic Cancer Cells Enhances NK-Mediated Cytotoxicity and Cytokine Production

**Figure 5:**
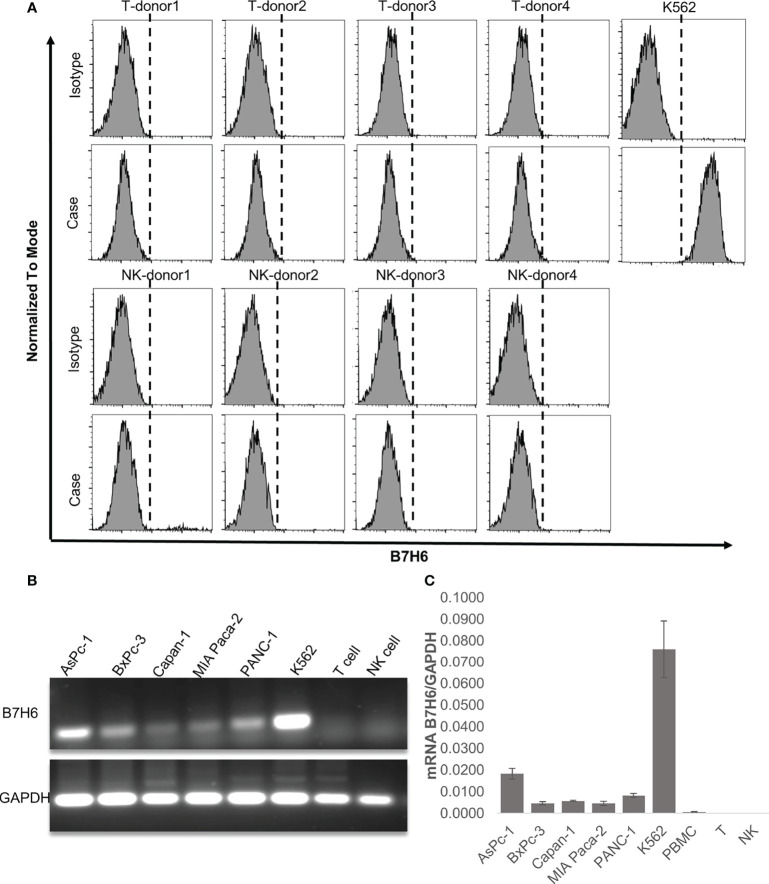
B7H6 expression in human immune cells. **(A)** Surface expression of B7H6 on human T cells or NK cells from 4 healthy donors was detected with a Fortessa X20 flow cytometer; the K562 cell line was a positive control. **(B)** Representative gel pictures of the B7H6 RT-PCR product from pancreatic cancer cell lines, human T cells, and NK cells; the K562 cell line was a positive control. **(C)** qPCR of B7H6 mRNA expression in human peripheral blood mononuclear cells, T cells, and NK cells. Five different PC cell lines were analyzed, and K562 cells served as the positive control. Data from at least 3 independent experiments are shown as mean ± SEM.

Our clinical data suggest that downregulation of B7H6 correlates with improved survival of PC patients. As shown above, however, it did not affect the characteristics of PC cells. Therefore, we speculated that B7H6 might impact immune cells that express its receptor, NKp30. To this end, we looked for surface expression of B7H6 on CD3^+^ T cells and CD56^+^ NK cells, using flow cytometry. These immune cells did not express B7H6 on their surface (**Figure 5A**). Moreover, we did not detect expression of B7H6 mRNA when we analyzed human T cells or NK cells with RT-PCR or qPCR (and used PC cells and K562 cells as positive controls) (**Figures 5B, C**). Next, we verified the expression of the B7H6 receptor NKp30 on human immune cells and other cancer cells. As expected, we found NKp30 exclusively on human NK cells and not on T cells or other human cancer cell lines (**Supplementary Figures 4A, B**). Consequently, we focused only on NK cells for the rest of the functional study.

**Figure 6:**
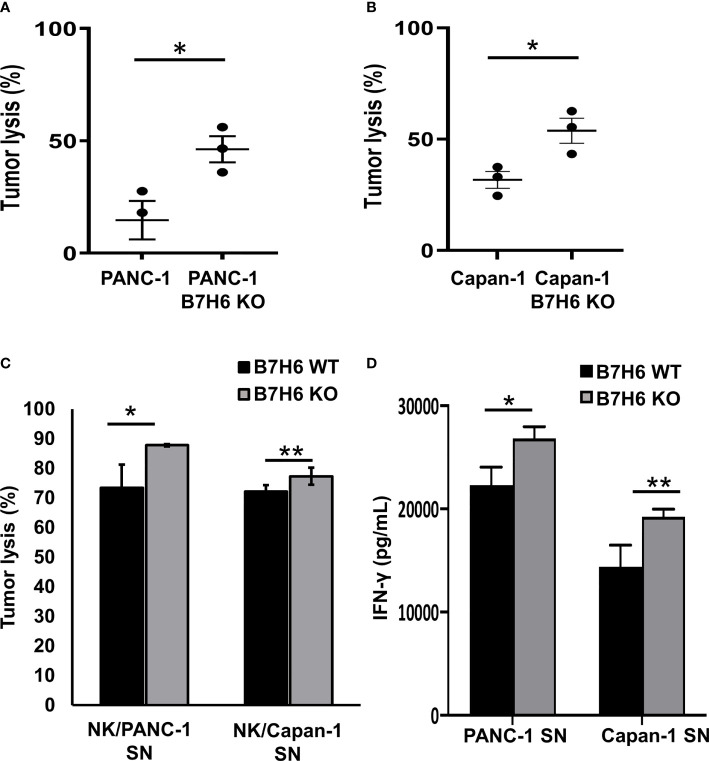
B7H6 mediates NK cytolytic functions. **(A, B)** Knocking out B7H6 enhanced NK-mediated tumor lysis on PANC-1 cells **(A)** or Capan-1 cells. **(B)** Analysis with an ACEA NovoCyte flow cytometer. Tumor lysis was calculated by: (cell counts from the tumor-only well − cell counts of the remaining living tumor cells in the sample well)/(cell counts from the tumor only-well − cell counts of the remaining living tumor cells in the well with lysis buffer). Living tumor cells were gated on the singlet, DAPI (-), and ZsGreen-labeled cells. The samples from each well all had the same volume. **(C)** Conditioned media collected from WT of PANC-1 and Capan-1 cells suppressed the tumor lysing function of NK cells compared to medium from B7H6 KO cells. Lysis was assessed with a standard 4-hour chromium^51^ release assay at an E/T ratio of 8:1. **(D)** IFN-γ released by NK cells that had been incubated in supernatant collected from either B7H6 WT or KO PANC-1 or Capan-1 cells. (*P < 0.05, **P < 0.01). The cytotoxicity assays were repeated with NK cells from 3 donors. Data are shown as mean ± SEM.

To investigate the potential impacts of B7H6 on NK cells, we co-cultured primary human NK cells with wildtype (WT) PC cell lines or PANC-1 and Capan-1 cells that lacked B7H6 (B7H6 KO cells). Compared to the WT cells of both PC cell lines, NK cells had significantly enhanced cytolytic activity when they targeted the B7H6 KO cells (**Figures 6A, B**). Furthermore, the low or absent expression of B7H6 in PC had no influence on the expression of HLA class I molecules in tumor cells (**Supplementary Figure 5**). Also, co-culture with B7H6 KO PC cells or WT PC cells had no effect on the expression of NK cell activating receptors (NKp30, NKG2D, and NKp46) or inhibitory receptors (NKG2A, PD-1 and TIGIT) (**Supplementary Figure 6**). Next, we incubated NK cells for 24 hours with supernatants collected from WT or B7H6 KO PC cells of PANC-1 and Capan-1 (PANC-1 SN and Capan-1 SN). The NK cells that had been incubated with supernatant from both types of B7H6 KO cells had significantly more cytolytic activity than those cultured with supernatant from the WT PC cells (**Figure 6C**). Blocking soluble B7H6 with anti-B7H6 antibody also enhanced cytolytic function compared to unblocked WT control. (**Supplementary Figure 7**). In addition, NK cells pre-incubated with B7H6 KO supernatant produced significantly more IFN-γ than those incubated with WT supernatant (**Figure 6D**). We also checked the expression of TNF-α, perforin, granzyme B, and CD69 in NK cells incubated with B7H6 KO supernatant. Expression of all those proteins was higher than with WT supernatant (**Supplementary Figure 8**). We knew that the supernatants contained soluble B7H6 because we used immunoblotting to check its basal level before the co-culture experiments (**Supplementary Figure 9**). Collectively, these findings led us to speculate that B7H6 is able to modulate NK cell cytotoxicity and cytokine production. Furthermore, sB7H6 may play a stronger role in our experimental system than cell-surface B7H6, as our data suggest that sB7H6 secreted by target cells may block the NKP30 receptor on NK cells. We hypothesize that soluble B7H6 can bind to NKp30 on NK cells. Masked in that way, NKp30 would be unable to bind to surface B7H6 on tumor cells, preventing NK cells from launching their lethal attacks on tumors.

## Discussion

B7H6, a new newly discovered member of the B7 family, has been found in many tumors but not in normal tissues. Moreover, expression of B7H6 in tumors correlates with tumor progression and poor patient survival—except in gastric carcinoma (14–22). To date, no studies of the clinical significance of B7H6 expression in human PC have been reported.

In this study, we demonstrated that B7H6 was expressed in 24 of 66 (36.4%) pancreatic cancer patient specimens. Its level on cancer tissues and in the tumor microenvironment strongly associated with tumor differentiation grade, lymphatic metastasis, and poor survival. Our study also showed that PC patients express two forms of B7H6—cell-surface B7H6 and soluble B7H6 (sB7H6). Both forms were upregulated in PC patients’ tissue or serum, and both associated with poor prognosis. Furthermore, sB7H6 was detectable in pancreatic cancer patients’ serum but not in that from healthy donors. Also, its level in serum correlated robustly with tumor differentiation grade and later TNM stage. In addition, a higher sB7H6 concentration indicated shorter overall survival of PC patients.

In *in vitro* experiments, all the pancreatic cancer cell lines expressed B7H6 mRNA and protein. Knocking out B7H6 in PC cells did not alter cell proliferation, apoptosis, or mobility. Instead, it enhanced NK-mediated cytolysis and cytokine production, suggesting that B7H6 helps modulate immune responses.

We used K562 cells as a positive control for B7H6 because they constitutively express B7H6 on their surface (cite). However, expression levels of B7H6 mRNA and protein levels in K562 cells were inconsistent with those in PC cells. This difference might be explained by post-transcriptional regulation in K562 cells, which requires further study.

B7H6 interacts with NK cells through the surface receptor NKp30, and the interaction plays an important role in NK cell-related immune responses (10). Sheffer et al. found that knocking out B7H6 from the surface of colorectal adenocarcinoma decreased the cytotoxicity of primary NK cells (38). Downregulation of cell-surface B7H6 expression on tumor cells also impaired tumor recognition by NK cells, helping tumor cells escape from NK cell-mediated killing (39). In contrast, Ponath et al. found that soluble B7H6 isolated from cell supernatant mediated a significant decrease in NK cell cytotoxicity (40). Moreover, Silvia et al. showed that the cytotoxic function of NK cells isolated from peritoneal/ascitic fluid of ovarian carcinoma patients was inhibited because NKp30 expression on NK cells was decreased by the high concentrations of sB7H6 (24). These paradoxical results indicate that B7H6 might have opposing functions in different cancers and that different forms of B7H6 might have opposite effects on NK cells. In the current study, we found that knocking out B7H6 increased cytokine production by NK cells and sensitized PC tumor cells to NK-mediated cytotoxicity. Thus in our experimental system, sB7H6 is more functionally dominant than the cell-surface form.

Some mechanistic studies have revealed how the different forms of B7H6 on NK cells function (41, 42). sB7H6 and sB7H6 and cell-surface B7H6 bind competitively to NKp30 on the NK cell surface (15); therefore sB7H6 could mask NKp30, making it unavailable to the B7H6 expressed on the surface of tumor cells (40). Moreover, a persistent receptor-ligand interaction between sB7H6 and NKp30 has been reported (24). Overall, the mechanisms of the opposing functions of B7H6 are still not clear. When we knocked out both cell-surface and soluble B7H6, co-culture with B7H6 KO tumor cells increased the anti-tumor function of NK cells. A similar result was obtained in the blocking experiment. Further studies are needed to distinguish the contributions of the two forms of B7H6 to PC.

## Conclusion

B7H6 appears to have clinical significance in various tumor types (43–47). In the current study, we demonstrated that it can serve as a negative prognostic marker in pancreatic cancer and that its downregulation makes PC tumor cells vulnerable to NK-mediated tumor lysis. In the future, it will be interesting to study the correlations between cell surface B7H6 and sB7H6 in a larger patient cohort. Further studies should also deepen our understanding of B7H6’s role during tumor progression and provide additional evidence that it could be a valuable target for diagnosis of PC and even for an anti-PC therapy.

## Data Availability Statement

The original contributions presented in the study are included in the article/**Supplementary Material**. Further inquiries can be directed to the corresponding authors.

## Ethics Statement

The studies involving human participants were reviewed and approved by First Affiliated Hospital of Soochow University. The patients/participants provided their written informed consent to participate in this study. Written informed consent was obtained from the individual(s) for the publication of any potentially identifiable images or data included in this article.

## Author Contributions

ZZ, K-YT, JZ, YX, XZ, LC, and DL conceived and designed the overall study. ZZ, K-YT, JZ, YX, XGZ, SH, LT, SM, ZLL, HZ, and JY contributed to the design of individual experiments. JZ, XZ, VC, and LZ provided critical reagents. ZZ, K-YT, YX, ZYL, TL, XC, XW, HC, ZD, and JW performed experiments. ZZ, K-YT, JZ, YX, and YP analyzed the data. WS and ZY helped analyze and interpret the pathology data. ZZ and K-YT wrote the manuscript with input from all co-authors. All authors contributed to the article and approved the submitted version.

## Conflict of Interest

The authors declare that the research was conducted in the absence of any commercial or financial relationships that could be construed as a potential conflict of interest.

## Publisher’s Note

All claims expressed in this article are solely those of the authors and do not necessarily represent those of their affiliated organizations, or those of the publisher, the editors and the reviewers. Any product that may be evaluated in this article, or claim that may be made by its manufacturer, is not guaranteed or endorsed by the publisher.

## Abbreviations

AML, acute myeloid leukemia; CPM, counts per minute; CRISPR, clustered regularly interspaced short palindromic repeats; FACS, fluorescence-activated cell sorting; NK cell, natural killer cell; PC, pancreatic cancer; qPCR, quantitative polymerase chain reaction; RT-PCR, reverse transcription-polymerase chain reaction; siRNA, small interfering RNA; TNM, Tumor, Node, Metastasis.
