# Supplementary material for: B7H6 Serves as a Negative Prognostic Marker and an Immune Modulator in Human Pancreatic Cancer
Source: Front Oncol. 2022 Mar 3;12:814312. doi: 10.3389/fonc.2022.814312 (PMC8929685; doi:10.3389/fonc.2022.814312)
Supplement: Supplementary file 1 [file DataSheet_1.pdf]

Supplementary Table 1 Clinical characteristics of 66 patients for Pancreatic cancer tissues

Characteristic	Number (%)	Characteristic	Number (%)
**Age (Years)**		**Differentiation grade**	
Median	68	Low	11(16.7)
Range	37–86	middle	51(77.2)
<68	31(47.0)	High	4(6.1)
≥68	35(53.0)	**Lymphatic metastasis**	
**Gender**		Yes	25(37.9)
Male	26(39.4)	No	41(62.1)
Female	40(60.6)	**TNM stages**	
**Tumor size (cm)**		I+II	29(43.9)
<2.0	20(30.3)	III+IV	37(56.1)
≥2.0	46(69.7)	**B7H6 expression**	
**Tumor location**		Positive	24(36.4%)
Head	41(62.1)	Negative	42(63.6%)
Body and tail	25(37.9)		

Supplementary Table 2 Clinical characteristics of 65 Pancreatic cancer patients for blood samples

Characteristic	Number (%)	Characteristic	Number (%)
**Age (Years)**		**Differentiation grade**	
Median	69	Low	20(30.8)
Range	45-84	middle	41(63.1)
<69	19(29.2)	High	4(6.1)
≥69	46(70.8)	**Lymphatic metastasis**	
**Gender**		Yes	31(47.7)
Male	38(58.4)	No	34(52.3)
Female	27(41.5)	**TNM stages**	
**Tumor size (cm)**		I+II	21(32.3)
<2.0	12(18.5)	III+IV	44(67.8)
≥2.0	53(81.5)	**B7H6 expression**	
**Tumor location**		ELISA>219.5	38(58.5%)
Head	43(66.2)	ELISA<=219.5	27(41.5%)
Body and tail	22(33.8)		

Supplementary Figure 1

A

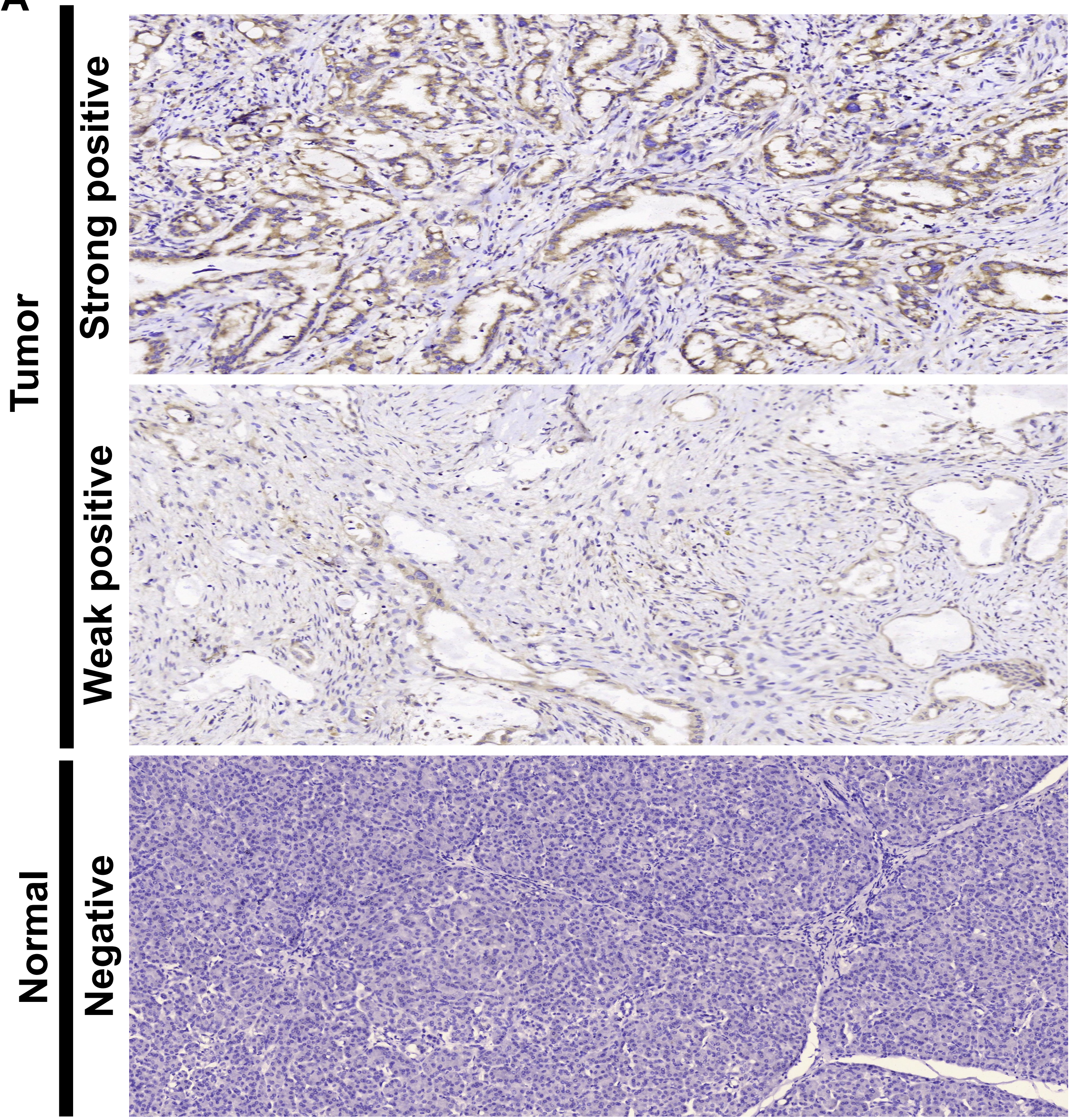

B

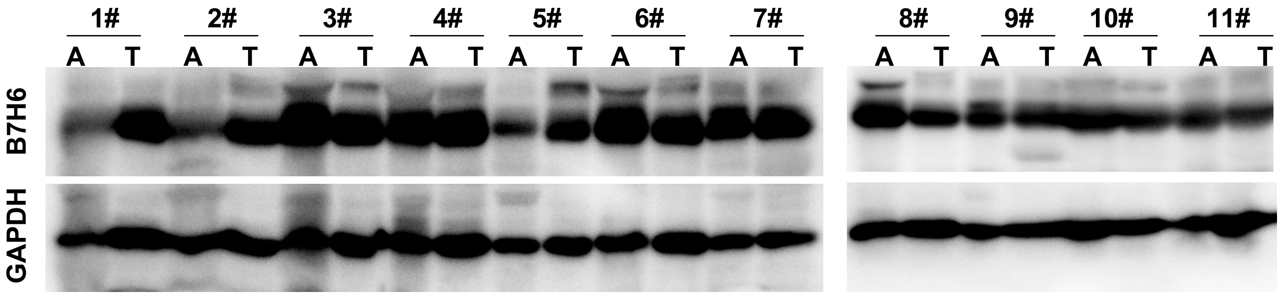

Supplementary Figure 1. Immunohistochemical staining for B7H6 in human PC tissues

(A) Representative images of high B7H6 expression in PC tissue (top), low B7H6 expression in PC tissue (middle), or negative B7H6 expression in normal Pancreas tissue (bottom). Original magnification was taken under 100X. **(B)** Immunoblotting analysis was performed to verify the protein expression of B7H6 in PC tumor tissues compared with the adjacent tissues (n=11). A: adjacent tissue, T: tumor tissue.

Supplementary Figure 2

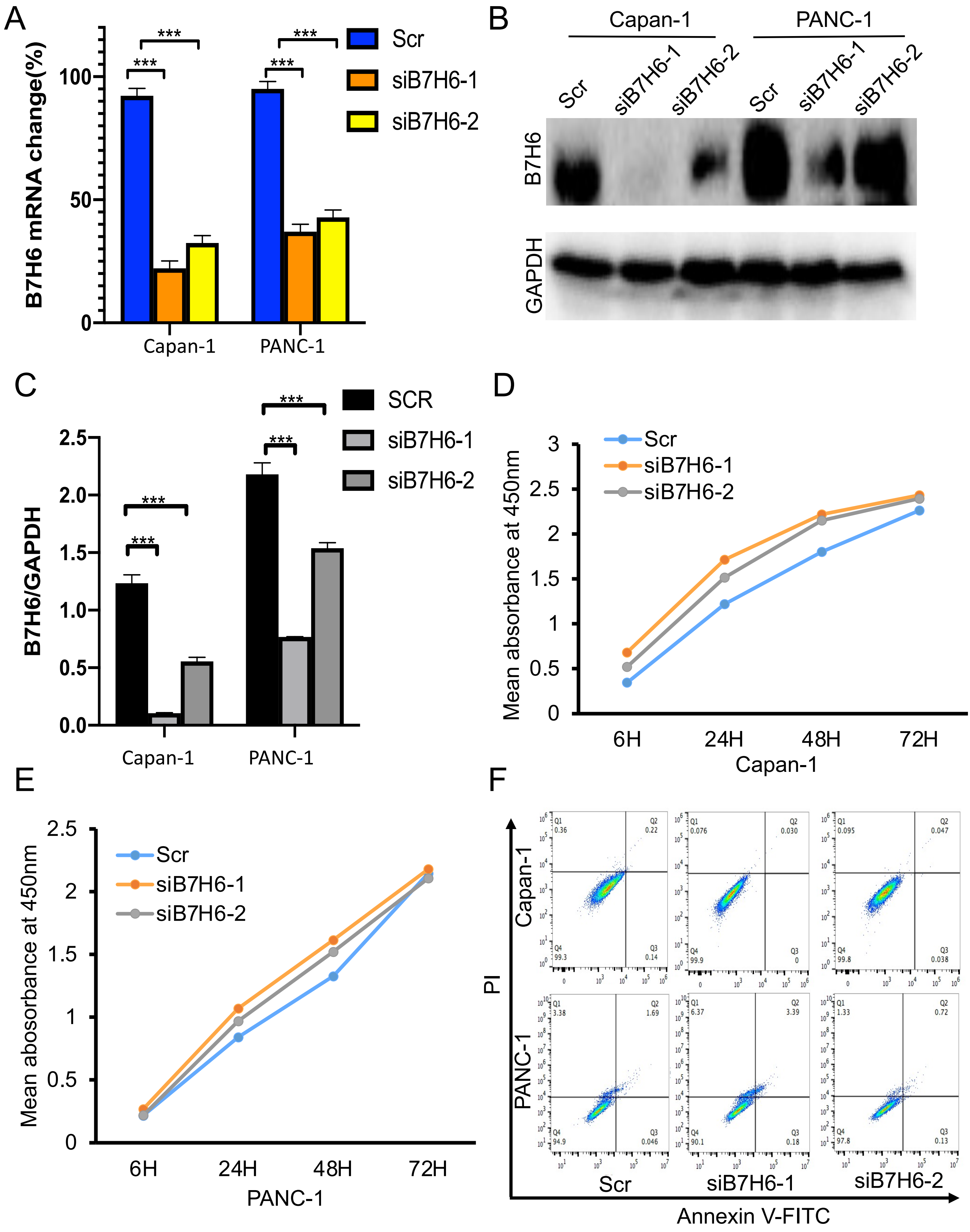

Supplementary Figure 2. B7H6 knockdown by siRNA in PANC-1 and Capan-1 cells

(A) The mRNA expression of B7H6 in the siRNA or scramble control groups from Capan-1 and PANC-1 cells analyzed by qPCR. **(B)** The protein expression of B7H6 knockdown by siRNA in Capan-1 and PANC-1 cells analyzed by immunoblotting. **(C)** The efficiency of B7H6 protein knockdown was quantified by software ImageJ. **(D)** Cell proliferation analysis by CCK-8 for Capan-1. **(E)** Cell proliferation analysis by CCK-8 for PANC-1. **(F)** Apoptotic Capan-1 and PANC-1 cells with B7H6 siRNA or negative control for 48h followed by flow cytometry after Annexin V-FITC/PI double staining. Scr: scramble control. Data are expressed as ± SD from at least 3 independent experiments.

Supplementary Figure 3

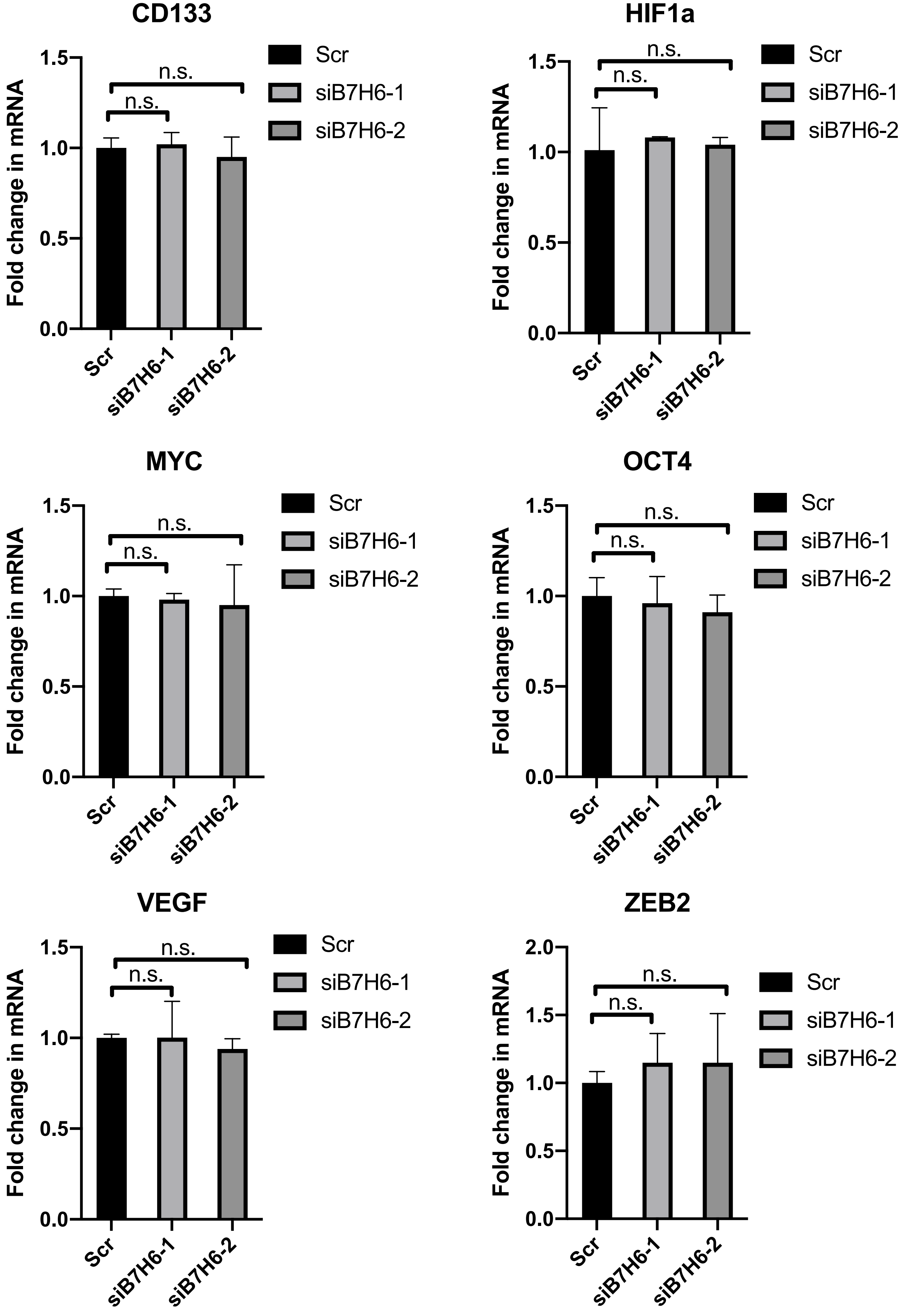

Supplementary Figure 3. B7H6 knockdown by siRNA did not affect the expression profile of tumor-associated genes in PC cells

Pancreatic cancer-associated genes including CD133, HIF1a, MYC, OCT4, VEGF and ZEB2 were analyzed by RT-qPCR in PC tumor cell line PANC-1. No significant differences were found between scramble control and B7H6 knockdown cells. Scr: scramble control , Data are expressed as ± SD from at least 3 independent experiments.

Supplementary Figure 4

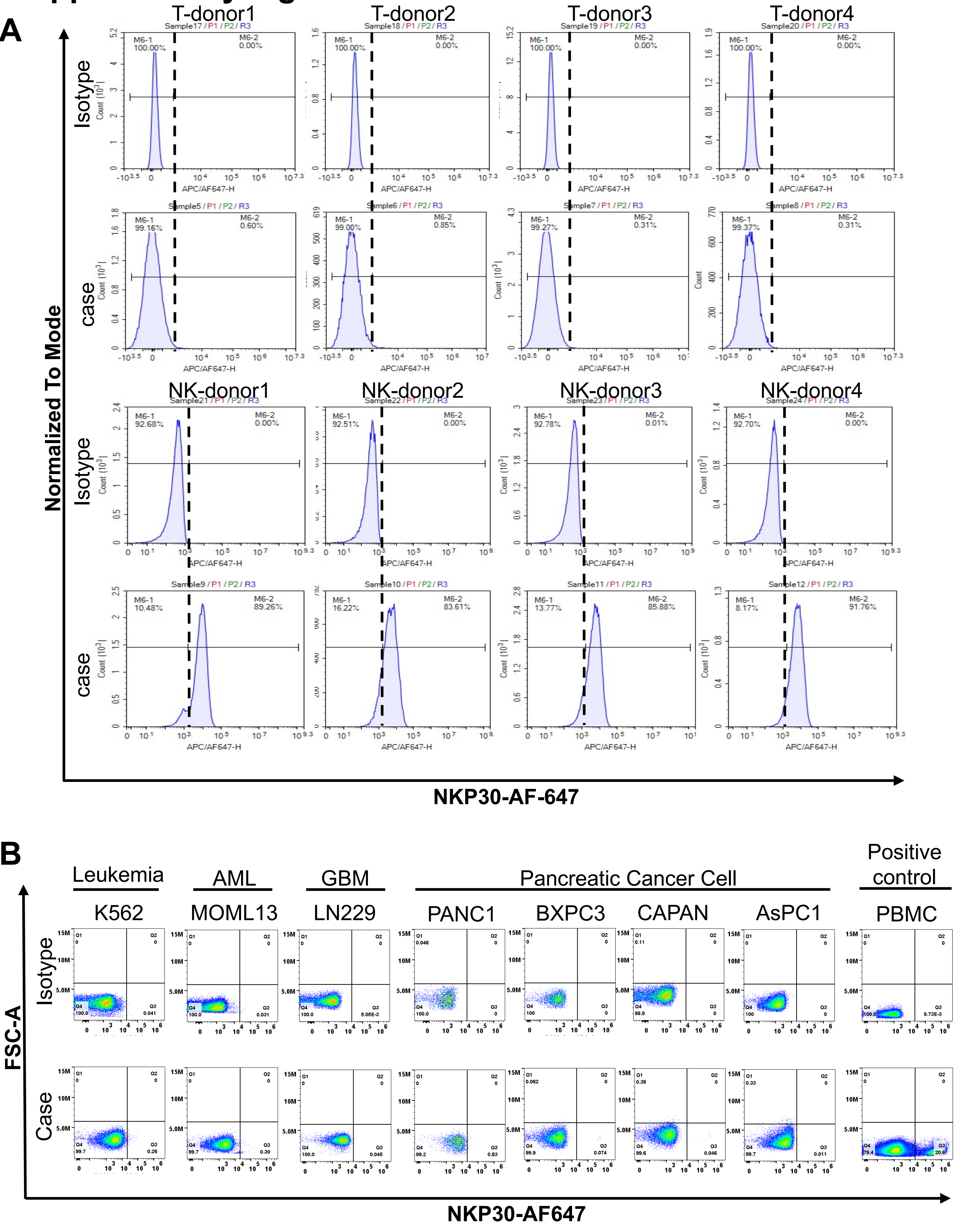

Supplementary Figure 4. The B7H6 receptor NKp30 expression in human immune cell and cancer cell lines

(A) Surface expression of the NKp30 on human T and NK cells from 4 different health donors were detected by flow cytometry.

(B) Surface expression of the NKp30 on K562 (CML), MOLM-13 (AML), LN229 (GBM), and Pancreatic cancer cells (PANC-1, BXPC3, Capan-1, AsPC-1 and mouse KPC cells) were detected by flow cytometry.

Supplementary Figure 5

A

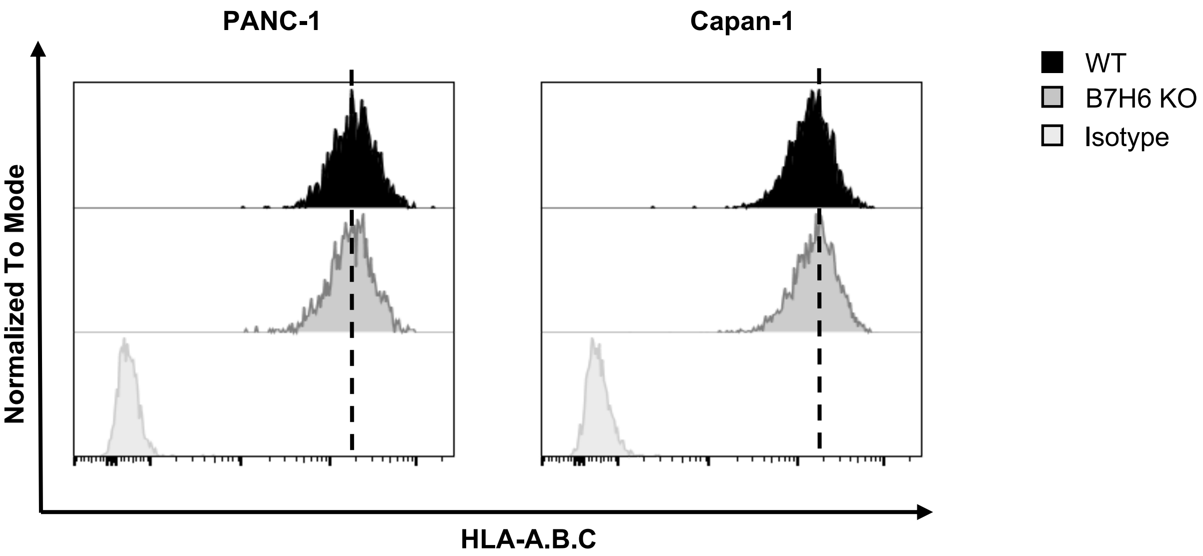

B

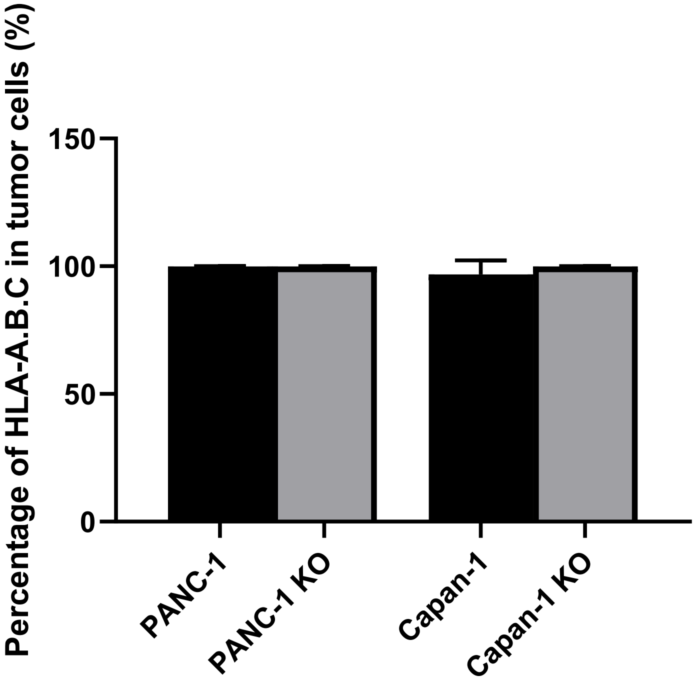

Supplementary Figur.5. HLA class I molecules HLA-A.B.C expression in human PC cell lines and the generation of B7H6 KO PC cell lines. A. Surface expression of the HLA-A.B.C on human Pancreatic cancer cell lines (PANC-1, CFPAC-1) assessed by flow cytometry. B. The quantity of the percentage of positive cells in the NK cells. Data are expressed as ± SD from at least 3 independent experiments.

Supplementary Figure 6

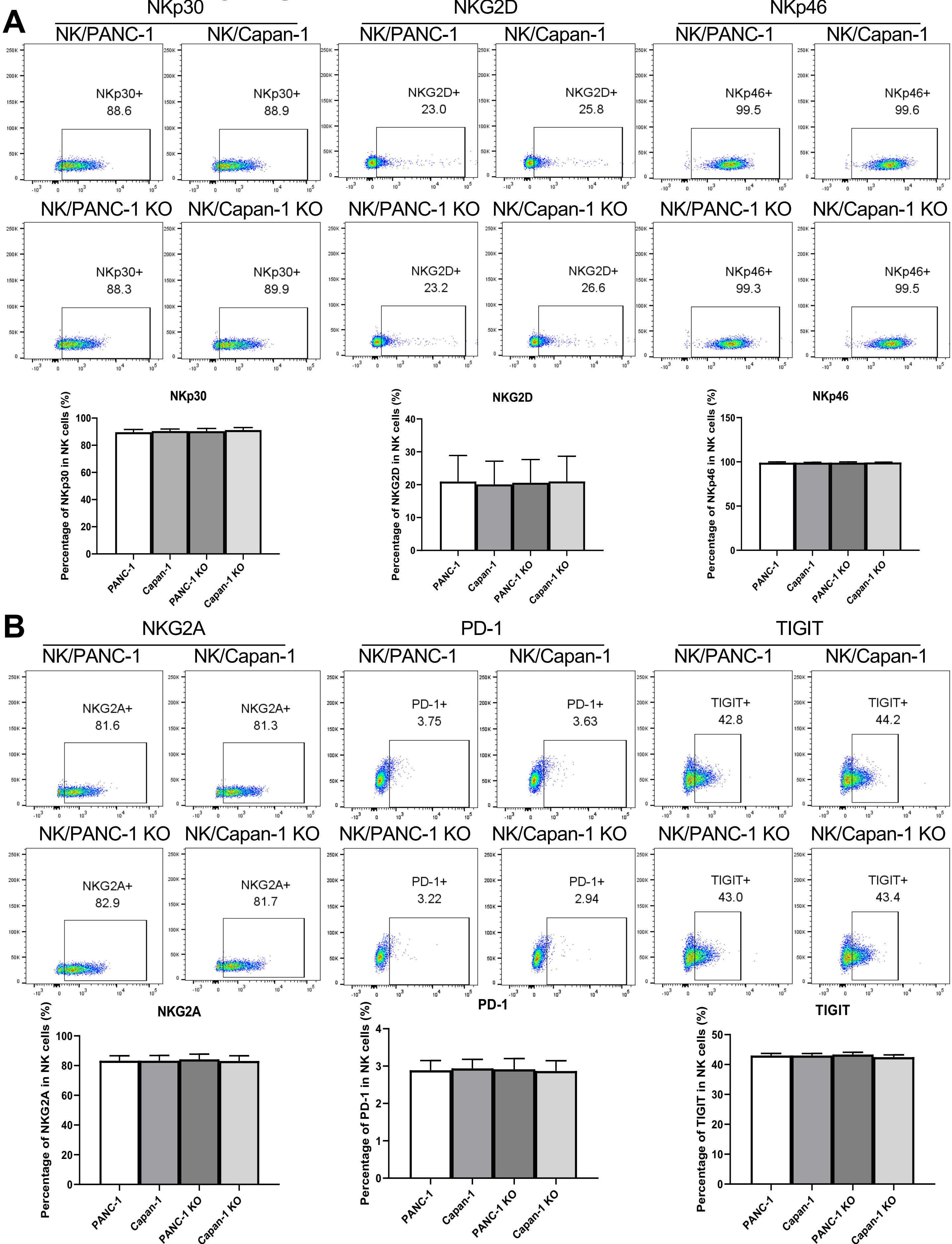

Supplementary Figure 6. Evaluation of activating and inhibitory receptors expression in the NK cells. NK cells co-cultured with PANC-1, Capan-1 and their B7H6 KO clones. The PANC-1 servers as B7H6-expressed control, the other cell lines servers as B7H6 low/absent cell lines. The activating receptors NKp30, PKG2D , NKp46 (sFig.5 A) and inhibitory receptors NKG2A, PD-1, TIGIT (sFig.5 B) been evaluated in NK cells by flow cytometric (top), and the expression shown by the percentage of positive cells are graphed at the bottom.(bottom). Data are expressed as ± SD from at least 3 independent experiments

Supplementary Figure 7

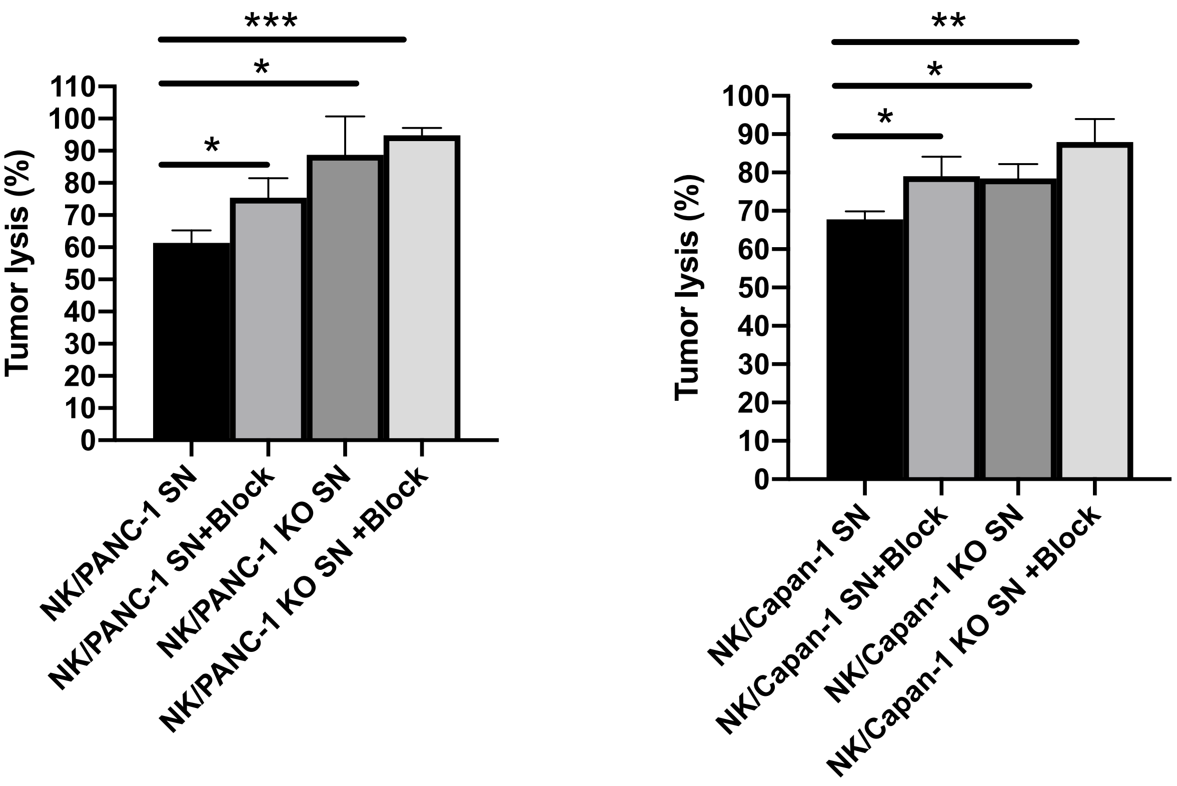

Supplementary Figure 7. Blocking assay for soluble B7H6 reacting with NK cells. The blocking antibody used as 100ng/ml in tumor supernatant for 40 minutes and co-cultured with NK cells overnight, them do the cytotoxicity assay by Cr51, Data are expressed as ± SD from at least 3 independent experiments.

Supplementary Figure 8

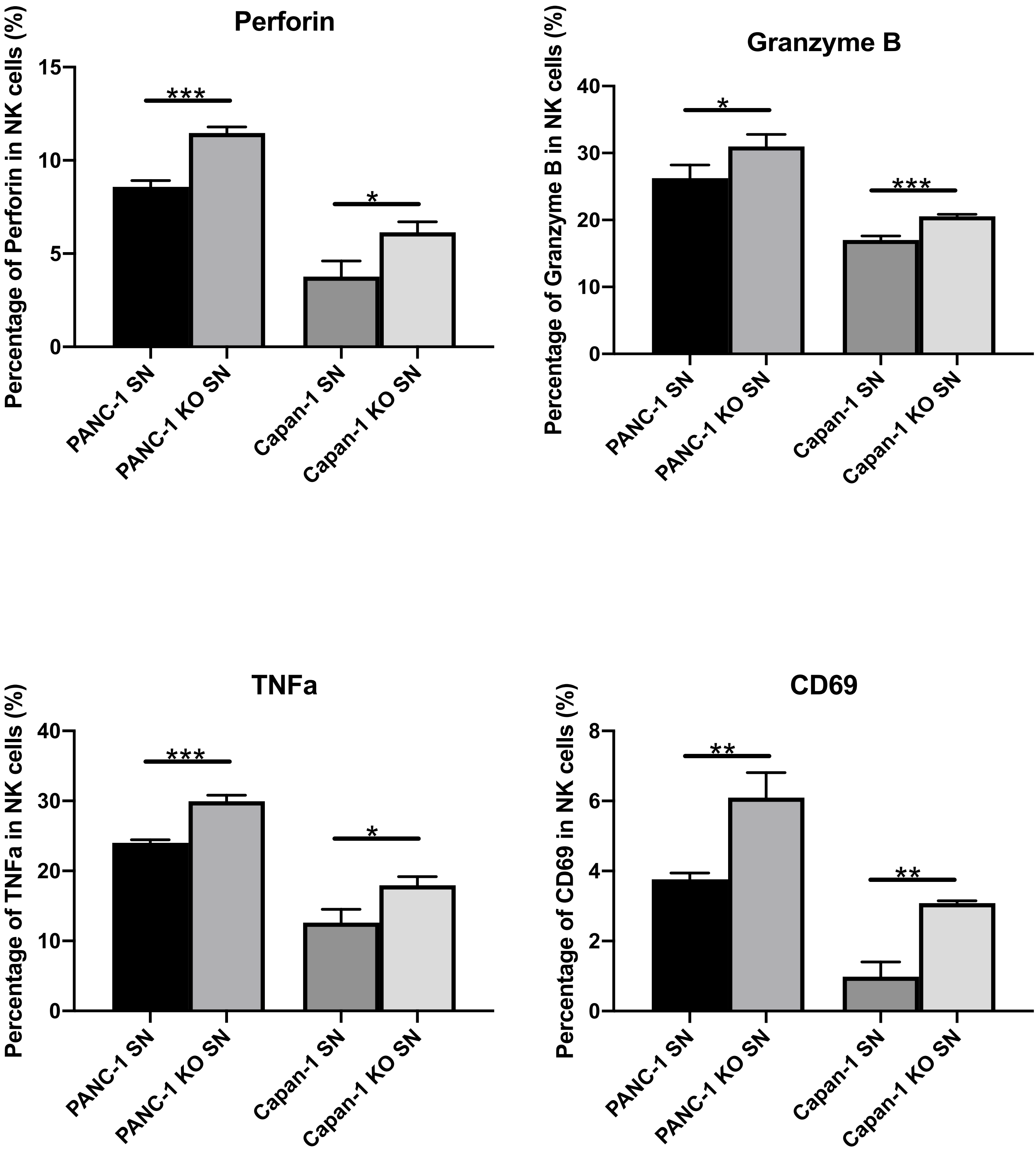

Supplementary Figure 8. Cytometric evaluation of perforin, granzyme B, TNF-α and CD69 expression in NK cells.. Primary NK cells cultured in the supernatant collected from wildtype PANC-1 and Capan-1 compared with B7H6 KO clones. Data are expressed as ± SD from at least 3 independent experiments.

Supplementary Figure 9

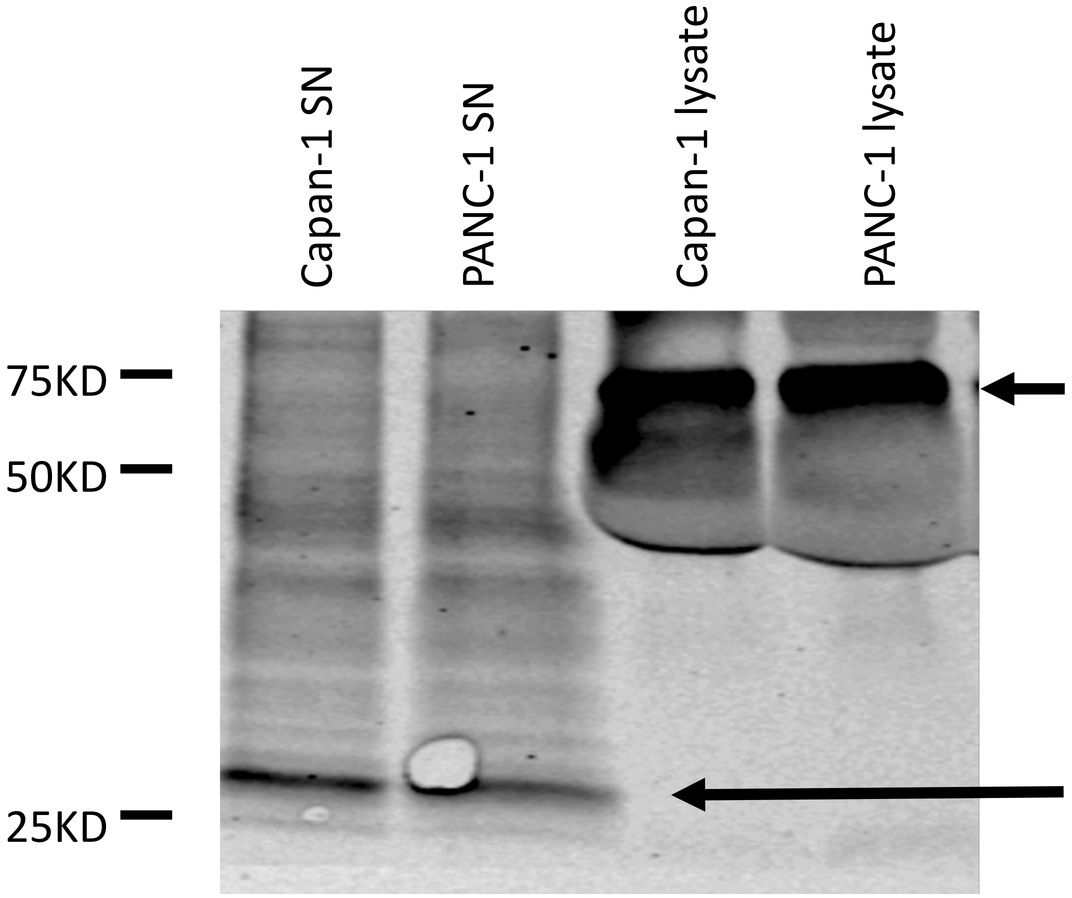

Supplementary Figur.9 B7-H6 expression in supernatants were analyzed by western blot:

Tumor cell lines were cultured for 48 hour and the supernatants were collected and analyzed by Western blot. The supernatants (SN) and lysates were prepared from Capan-1 and PANC-1 cells were analyzed by immunoblot with Abs specific for B7-H6. Arrows indicate the sizes of full length B7-H6 (55 kDa) and soluble B7-H6 (30 kDa).
